# Logistic analysis of delayed reporting of emergency blood potassium and comparison of improved outcomes

**DOI:** 10.1038/s41598-024-56667-1

**Published:** 2024-03-13

**Authors:** Jian Zhang, Shuangshuang Lv, Tingting Jin, Xiaxuan Hu

**Affiliations:** Clinical Laboratory, Dongyang People’s Hospital, No. 60 Wuning West Road, Dongyang City, 322100 Zhejiang China

**Keywords:** Emergency service hospital, Potassium, Multivariate analysis, Emergency medicine, Centrifugation, Risk factors, Medical research, Risk factors

## Abstract

Potassium testing is an essential test in emergency medicine. Turnaround time (TAT) is the time between specimen receipt by the laboratory and the release of the test report. A brief in-laboratory TAT increases emergency department effectiveness. Optimizing processes to shorten TAT using other tools requires extensive time, resources, training, and support. Therefore, we aimed to find a convenient way to shorten TAT, identify risk factors affecting the timeliness of emergency potassium test reporting, and verify the intervention’s effects. The dependent variable was emergency potassium reporting time > 30 or < 30 min. Logistic analysis was performed on monitorable factors, such as sex, age, potassium results, number of items, specimen processing time (including centrifugation and time before specimen loading), critical value ratio, instrument status, shift where the report was issued, specimen status, and work experience, as independent variables. In the multivariate analysis, work experience, instrument failure rate, and specimen processing time were risk factors for emergency blood potassium reporting exceeding 30 min. Improvement measures were implemented, significantly decreasing the timeout rate for acute potassium reporting. Our study confirms the usefulness of logistics in reducing the time required to report potassium levels in the emergency department, providing a new perspective on quality management.

## Introduction

The potassium test is an important and commonly used test in emergency medicine to assess vital signs and functional status. Blood potassium levels are essential for the proper functioning of the heart, nerves, and muscles. Acute abnormalities in serum potassium, such as hyperkalemia or hypokalemia, can cause serious problems, such as arrhythmias^1–3^ and muscle weakness^4^. In the emergency department, patients with critical conditions, such as heart attacks and serious infections, are often treated. Hence, rapid potassium testing can help doctors assess the patient's vital signs and functional status, allowing for the timely detection and treatment of abnormal serum potassium and ensuring the patients’ safety and health^5^. Additionally, potassium testing can guide disease diagnosis and treatment decisions, as abnormal serum potassium levels may be associated with various conditions and drug use^6,7^. For example, hyperkalemia can be associated with conditions such as renal failure^8^ and diabetic ketoacidosis^9^, while hypokalemia may be associated with conditions such as diuretic use, vomiting, or diarrhoea^4,10^. For patients with abnormal serum potassium, regular testing of blood potassium levels allows doctors to assess the treatment’s effectiveness and make timely adjustments to drug doses or treatment regimens. Serum potassium levels are also important for patients’ prognosis. Hospitalization due to hypokalemia is associated with adverse outcomes in vasospasmodic angina^2^, and severe electrolyte disturbances are significantly associated with neurological morbidity and mortality^11^. Therefore, timely and accurate blood potassium reporting is significant for physicians' decision-making and patient treatment.

The turnaround time (TAT) in the laboratory is the time between the receipt of the specimen by the laboratory and the release of the test report. This concept helps measure the timeliness of laboratory reporting and provides guidance for improving reporting delays^12^. A short in-laboratory TAT means doctors can obtain laboratory reports promptly. This is particularly crucial in emergency departments with challenges such as high workloads, tight schedules, and diverse disease varieties. Shorter reporting times can improve productivity and reduce patient wait times, positively impacting emergency department operations and patient outomes^13–16^.

The TAT in the laboratory is influenced by various factors^17–20^. First, the performance of laboratory equipment and instruments is directly related to the inspection condition^18^. Second, the adequacy of human resources also impacts inspection TAT. Understaffed laboratories with workloads greater than the capacity of their human resources will inevitably experience decreased inspection efficiency and reporting delays. In addition, optimizing processes and management is an essential factor affecting emergency examinations TAT^21,22^. Properly designed and effectively managed laboratory processes help reduce unnecessary time waste and improve inspection efficiency.

Traditionally, quality control circles, Plan-Do-Check-Act, and other quality management tools are used to optimize and improve processes and management to shorten TAT. However, these methods require significant time, resources, training, and support, leading to limitations and variable results. We aimed to find a simpler and more convenient way to improve the timeliness of emergency blood potassium reporting. Therefore, we retrospectively quantified the factors affecting the timeliness of emergency blood potassium reporting and used logistic regression analysis to find and improve the risk factors affecting the timeliness of reporting. The final intervention effect confirmed the effectiveness of logistics in shortening the time for emergency blood potassium reporting, offering a new perspective for quality management.

## Methods

### Background information

**Figure 1:**
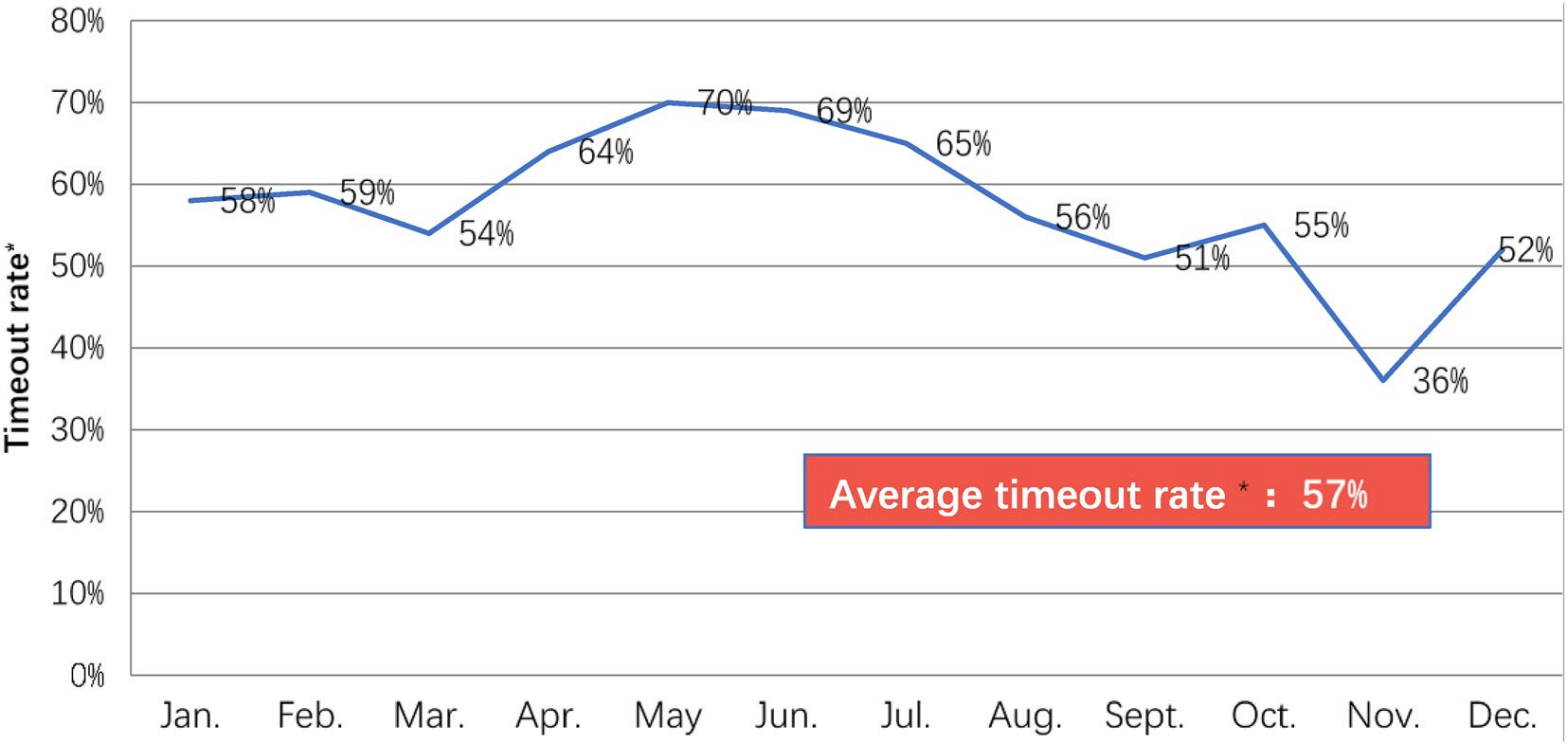
Analysis of the timeout rate of 30 min of blood potassium reports in 2022.

**Figure 2:**
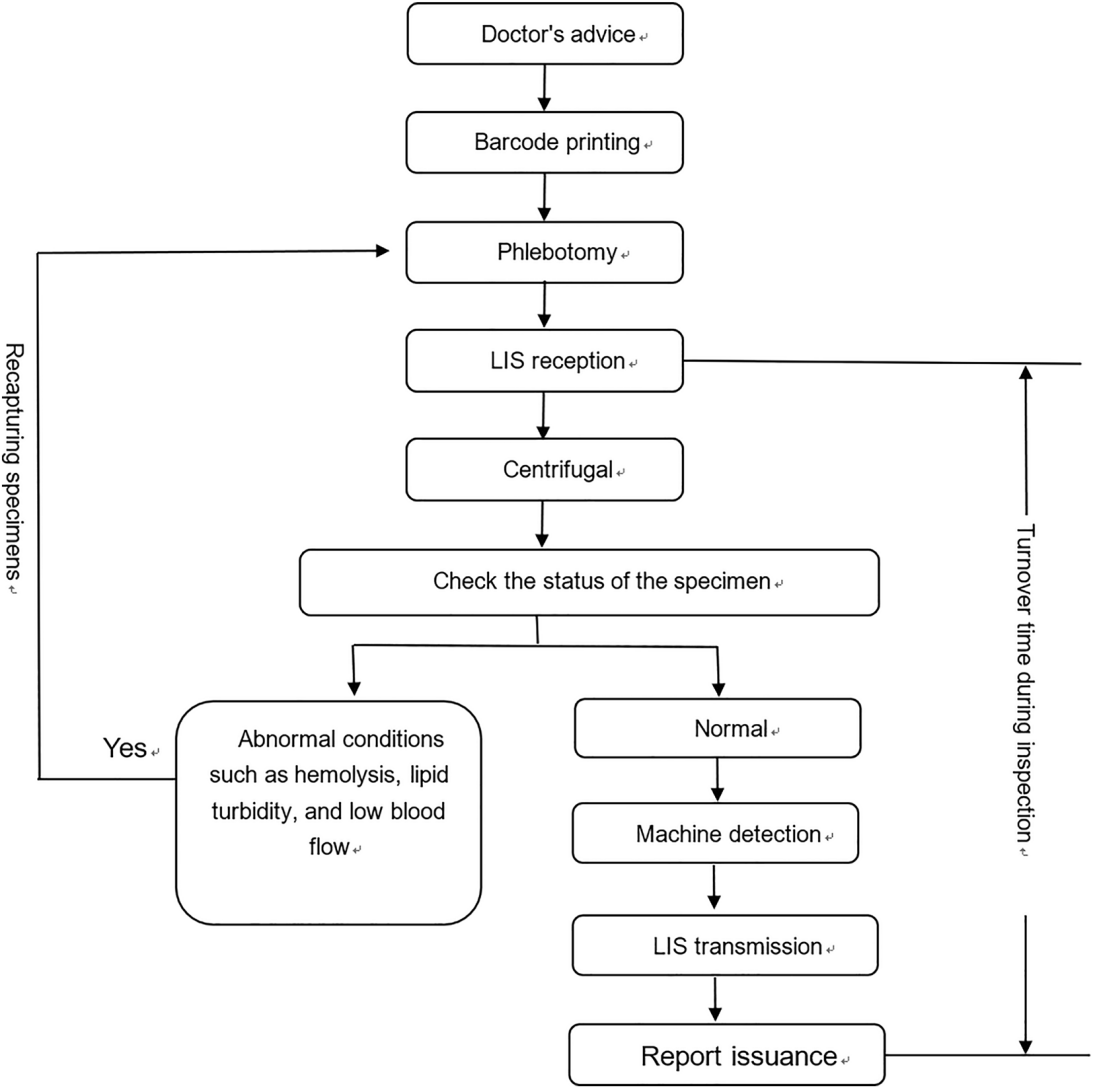
Emergency blood potassium sample flowchart.

According to the regulations of the Zhejiang Provincial Medical Security Bureau, the proportion of emergency potassium test reporting time exceeding 30 min must be within 5%. However, in our hospital, the proportion of emergency potassium reporting time exceeding 30 min in 2022 was, on average, 57%, far exceeding this requirement, as shown in Fig. 1. We listed the process of emergency potassium testing and the monitorable risk factors in each process and addressed the risk factors for each (Fig. 2).

The study was conducted at a 1700-bed general tertiary hospital in China. The emergency laboratory obtained ISO 15189 accreditation in 2014. We selected 9873 potassium reports issued by the laboratory from April to May 2022 as pre-intervention data. Information was collected through the Laboratory Information Management System (LIS), including TAT, reception time, machine testing time, report sending time, number of tests in the project, work experience, results, specimen status, and instrument failure rate for each laboratory potassium specimen. In total, we collected 13,984 potassium reports for the same period in 2023 as post-intervention data. Inclusion criteria: the selected reports included potassium tests issued by the laboratory, including individual potassium tests and emergency combination tests. Studies in which the reporting time was more than 24 h and the report failed to be sent for various reasons were excluded from the analysis. All samples were tested using the Cobas c 501 module and original matching reagents (Roche, Switzerland). After the intervention, the two Cobas c 501 modules for clinical chemistry were backed up (one for the day shift and the other for the night shift). This study was noninterventional. Data for all patients were obtained through retrospective retrieval and were anonymized for analysis. The study was approved by the institutional review board (IRB) of Dongyang People's Hospital. Since this was a retrospective study, the IRB of Dongyang People's Hospital waived the requirement for informed consent of the study participants. All methods of this study were performed in accordance with the relevant guidelines and regulations.

### Analysis of influencing factors

**Figure 3:**
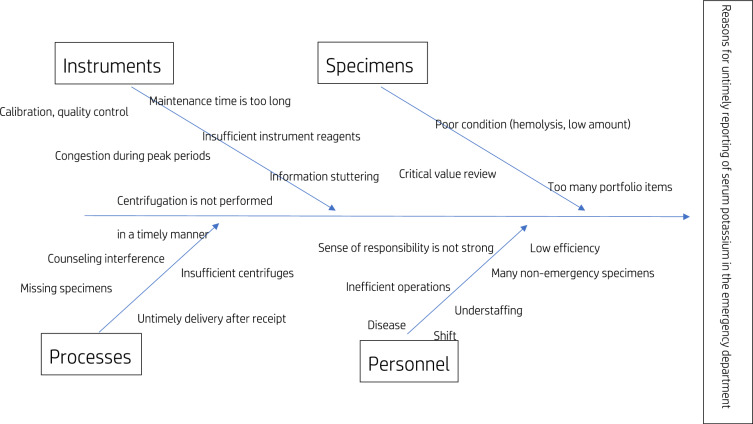
Fishbone diagram of the causes of untimely reporting of serum potassium in the emergency department.

We organized four resident staff members in the emergency laboratory with qualifications equivalent to the chief laboratory technician or above to create a fishbone diagram of the causes for the delay in reporting potassium (Fig. 3). We combined those data with previous findings and reports^19,22^. We quantitatively analyzed the risk factors from four aspects: personnel, instruments, processes, and specimens.

In terms of personnel, we categorized all personnel capable of sending blood potassium reports into seven groups according to their work experiences: Group A had ≥ 30 years of service; Group B had 25–30 years of service; and subsequent groups with a decreasing range of 5 years, ending with Group G with < 5 years of service.

In terms of instruments, we counted failures of centrifuges, biochemical testing instruments, and computer systems. The instrument failure rate is the amount of blood potassium received during the biochemical detector and computer malfunctions divided by the total amount of blood potassium.

In terms of process, we recorded the specimen processing time (from specimen receipt to the time before instrument testing), the shift when the report was released, and the specimen receiving time.

In terms of specimens, we counted the results of the potassium test, the number of items containing potassium, the proportion of critical values, and the condition of the specimens. According to the fourth edition of the Clinical Laboratory Center guidelines issued by the Ministry of Health of China, the normal serum potassium level is 3.5–5.5 mmol/L. Serum potassium levels below 2.8 mmol/L or above 6.2 mmol/L are considered critical values. The critical value ratio is the number of critical values divided by the total number of serum potassium reports.

### Logistic analysis

The timeout rate of the potassium test was reported as a dependent variable, with reporting times categorized as 0 for under 30 min and 1 after 30 min. Independent variables included sex, age, length of service of the employee, instrument status, specimen processing time, shift when the report was issued, time of specimen receipt, results of the potassium test, number of potassium in the project portfolio, critical value ratio, and specimen status. In this context, women were defined as 0 and males as 1. The report is published during the following shifts: Day Shift (8:01–11:30 and 13:31–17:00) = 0, Middle Shift (11:31–13:00) = 1, Night Shift 1 (17:01–22:00) = 3, and Night Shift 2 (22:01–8:00) = 4. Reported years of experience: A (30 or more years of experience) = 0, B (25–30 years of experience) = 1, C (20–25 years of experience) = 2, D (15–20 years of experience) = 3, E (10–15 years of experience) = 4, F (5–10 years of experience) = 5, and G (under 5 years of experience) = 6. Non-critical value = 0, Critical value = 1; Instrument operating status: normal = 0, fault = 1. Serum potassium specimen status: normal = 0, hemolysis = 1, blood deficiency = 2, and fat turbidity = 3 (Table 1).

**Table 1:** Assignments and variables of overtime factors in emergency blood potassium testing.

Name	Meaning	Assignment
Y-TAT	Outcome variable, the time when the sample was received in the laboratory and the report was sent out	TAT ≤ 30 min = 0; TAT > 30 min = 1
X-sex	–	Female = 0; Male = 1
X-age	–	Continuous variable, in years
X-results	–	Continuous variable, in mmol/L
X-test	Number of tests included in the same medical order	Continuous variable, in terms
X-shift	The time period during which the report was issued	Day shift (8:01–11:30 and 13:31–17:00 = 0; Middle shift (11:31–13:00) = 1; Late night shift 1(17:01–22:00) = 3; Late night shift 2 (22:01–8:00) = 4
X- experience	Working years	A(30 years or more of working experience) = 0; B(25 to 30 years of experience) = 1; C(20 to 25 years of experience) = 2; D(15 to 20 years of experience) = 3; E(10 to 15 years of experience) = 4; F(5 to 10 years of experience) = 5; G(Under 5 years) = 6
X-critical	Less than 2.8 mmol/L or greater than 6.2 mmol/L^※^	Non-critical value = 0; Critical value = 1
X-wait	The time before the specimen is received and tested on the machine	Continuous variable, in seconds
X-instrument	Instrument operation status	Normal = 0, Faulty = 1
X-status	Status after specimen collection	Normal = 0; Hemolysis = 1; Blood deficiency = 2; Fat turbidity = 3

TAT, turnaround time; ^※^, from the fourth edition of the Clinical Laboratory Center of the Ministry of Health issued by the Ministry of Health of China.

### Interventions

In light of the multivariate analysis results, the following steps were taken to address delays.

1. Personnel: Building on insights from the staff training program and a previous report^23^, we adjusted the training plan for employees within 5 years. The original 3-month emergency laboratory rotation period was extended to 1 year, and a training plan was formulated. This plan outlined the training content of the emergency laboratory rotation personnel for the first week, the first month, and the first quarter of the assessment, including the operation of various instruments in the emergency laboratory and report processing. Further, to increase the attention of employees to the TAT of blood potassium reporting, we published the potassium test TAT of each employee group from the previous month at the beginning of each month. Additionally, we implemented a reward system for those who reported the lowest overtime rate in the current month.
2. The instrument failure rate was high, including problems with biochemical instruments, centrifuges, and computer software. Hence, we purchased a biochemical analyzer and centrifuge. The two biochemical instruments are now used alternately during the day and night to prevent continuous operation. The two centrifuges run simultaneously to meet the continuous receipt of blood potassium specimens. Additionally, in cooperation with information technicians, the computer information system of the emergency laboratory was upgraded to reduce information congestion.
3. The waiting time for specimens was undesirably long, encompassing the time from specimen receipt at the laboratory to its testing on the machine, including the centrifugation and transportation stages. To reduce the centrifugation phase, we added a second centrifuge, allowing prompt centrifugation of blood specimens after phlebotomy. To reduce the time of the specimen transport phase, we set up an audible timer on the centrifuge, prompting staff to load the next specimen promptly. Additionally, due to the outbreak of respiratory diseases in recent years, emergency laboratories often experience a surge in respiratory specimen testing; therefore, we added emergency personnel to deal with the surge in other specimens (Table 2).

**Table 2:** Analysis of the reasons for delayed emergency blood potassium reporting and implementation of countermeasures.

Factor	Reason	Countermeasure
Experience		
	Young employees have low work efficiency	Increase operational training and extend emergency shift time if necessary
	Not valuing TAT	Regularly publish TAT data and establish a reward system
High instrument failure rate		
Biochemical instruments	Single biochemical instrument with long maintenance time; 24-h uninterrupted use with high failure rate	Add a standalone emergency biochemical instrument
Computer system	Information stuck, system malfunction	Updating and improving the laboratory information system
The waiting time for the specimen is too long		
Centrifugal stage	Only one centrifuge	Purchase a centrifuge
Transport phase	Delayed internal transportation	Set timed reminders
Externalities	Respiratory disease outbreaks, the workload is heavy	Add emergency positions

*TAT* turnaround time.

### Statistical analyses

SPSS statistical software (version 26.0; IBM Corp., Armonk, NY, USA) was used for statistical analysis and processing. Normally distributed continuous variables are represented as means and standard deviations. Non-normally distributed data are represented as medians and interquartile ranges (IQR). Chi-square tests were used to compare categorical variables. We performed the Student's t-test and Wilcoxon's rank test to compare parametric and nonparametric continuous variables, respectively. Logistic regression analysis was performed using whether or not the time for the potassium test exceeded 30 min as the dependent variable and other factors as independent variables. The variable assignment is shown in Table 1. P values < 0.05 were considered statistically significant.

## Results

### Logistic analysis results

In cases where the value of each factor is 0, indicating that the factor is at the baseline level and the Exp(B) value is positive, the report is considered delayed.

Univariate analysis showed that factors associated with a statistically significant delay in emergency potassium reporting (P < 0.05) were the number of portfolio tests, specimen waiting time, critical value ratio, instrument failure rate, specimen status, work experience, and reporting shift.

Multivariate analysis showed that the factors influencing delays in reporting potassium in the emergency department were work experience (P < 0.05), instrument failure rate (P < 0.05, odds ratio [OR] = 1.46), and specimen processing time (P < 0.05, OR = 1.13). Regarding work experience, group G was statistically significant. Compared to group A (group A = 0), group G (P < 0.05, OR = 14.47) was more likely to be associated with reporting delays (Table 3).

**Table 3:** Logistic analysis of factors affecting emergency blood potassium timeout.

Factor	Timeout group (n = 6602)	Not timeout group (n = 3271)	Univariate analysis			Multivariate analysis		
	TAT > 30 min	TAT ≤ 30 min	Exp (B)	P	OR (95% CI)	Exp (B)	P	OR (95% CI)
Sex (F/M)	2817/3785	1411/1860	0.02	0.66	1.02 (0.94–1.11)	–	–	–
Age	52.10 ± 24.32	51.37 ± 24.17	0.00	0.16	1.00 ((1.00–1.00)	–	–	–
Results	3.91 ± 0.58	3.90 ± 0.48	0.04	0.34	1.04 (0.96–1.12)	0.42	0.14	1.53 (0.88–2.66)
Sample receiving time	13.07 ± 6.19	13.12 ± 5.09	0.00	0.73	1.00 (0.99–1.00)	− 0.07	0.20	0.93 (0.83–1.04)
Test	8.68 ± 3.10	7.80 ± 3.47	0.08	0.00**	1.08 (1.07–1.10)	− 0.09	0.05	0.91 (0.84–0.99)
Wait	2696.06 ± 1000.05	1552.44 ± 202.48	0.10	0.00**	1.11 (1.09–1.12)	0.13	0.00**	1.13 (1.11–1.16)
Critical value ratio	4.32%	9.17%	1.58	0.00**	4.88 (3.34–7.12)	− 0.06	0.97	0.94 (0.04–21.09)
Instrument failure rate	67.57%	0.27%	6.63	0.00**	755.19 (391.79–1455.67)	0.38	0.04*	1.46 (0.52–4.11)
Day shift	2860	1138		0.00**	1.00 (reference)		0.79	1.00 (reference)
Middle shift	1174	545	0.73	0.00**	2.07 (1.81–2.35)	− 0.19	0.65	0.83 (0.36–1.90)
The night before shift	1872	1016	0.57	0.00**	1.77 (1.52–2.06)	0.45	0.40	1.56 (0.55–4.46)
Late night shift	696	572	0.41	0.00**	1.51 (1.32–1.73)	− 0.47	0.49	0.63 (0.16–2.41)
Sample status: Normal	5537	2997		0.00**	1.00 (reference)		0.90	1.00 (reference)
Sample status: hemolysis	721	9	3.85	0.00**	47.15 (24.40–91.12)	0.12	0.84	1.13 (0.37–3.45)
Sample status: Low blood volume	145	2	3.75	0.00**	42.67 (10.56–172.37)	− 1.08	0.51	0.34 (0.01–8.43)
Sample Status: Fat Turbid	199	1	4.76	0.00**	117.13 (16.41–836.00)	− 0.59	0.74	0.55 (0.02–19.40)
Experience A	199	80		0.00**	1.00 (reference)		0.00**	1.00 (reference)
Experience B	215	143	0.05	0.71	1.05 (0.81–1.37)	0.67	0.55	1.95 (0.22–17.50)
Experience C	102	42	− 0.40	0.00**	0.67 (0.54–0.83)	0.20	0.88	1.22 (0.09–17.04)
Experience D	1428	742	− 0.02	0.91	0.98 (0.69–1.40)	0.27	0.75	1.31 (0.26–6.70)
Experience E	326	202	− 0.16	0.00**	0.85 (0.76–0.95)	− 0.14	0.90	0.87 (0.11–6.74)
Experience F	949	511	− 0.36	0.00**	0.70 (0.58–0.84)	− 0.29	0.73	0.75 (0.14–3.93)
Experience G	3383	1551	− 0.24	0.00**	0.79 (0.70–0.89)	2.67	0.00**	14.47 (2.83–73.92)

TAT, turnaround time; Text, the number of tests included in the same report; Wait, the time before the specimen is received for on-machine testing; OR, Odds ratio; CI, confidence interval; **P < 0.01; *< 0.05; bold value is a factor that needs to be corrected in this study.

### Post-intervention outcomes

After implementing the above improvement measures, the results indicated that the TAT (IQR) of the emergency blood potassium laboratory decreased from 29 (35–56) min to 21 (23–32) min. The overtime rate for emergency blood potassium reports decreased from 66.9 to 11.8%. The sample processing time was reduced from 2317.2 ± 985.9 s to 1507.7 ± 535.2 s. The instrument failure rate decreased from 45.3 to 0.1%. The overtime rate of employees decreased significantly; for example, the overtime rate for group G reporting dropped from 69.2 to 11.7% (P < 0.05) (Table 4).

**Table 4:** Comparison of various factors before and after intervention.

	Before (n = 9,873)	After (n = 13,984)	X^2^/t	P
TAT (min)	29 (35, 56)	21 (23, 32)	93.9	0.00**
30 min timeout rate (%)	66.9%	11.8%	7754.7	0.00**
Instrument failure rate (%)	45.3%	0.1%	7722.9	0.00**
Specimen waiting time (s)	2317.2 ± 985.9	1507.7 ± 535.2	81.6	0.00**
Report timeout ratio for each group of employees, %(N)				
A	70.3% (196)	10.9% (200)	558.3	0.00**
B	60.1% (215)	16.1% (20)	71.1	0.00**
C	68.8% (99)	14.7% (5)	33.1	0.00**
D	65.6% (1,424)	10.4% (206)	1,323.6	0.00**
E	61.0% (322)	9.4% (93)	462.4	0.00**
F	63.8% (932)	16.4% (242)	689.6	0.00**
G	69.2% (3,414)	11.7% (885)	4,363.5	0.00**

TAT, turnaround time; Specimen waiting time, the time it takes for the specimen to be received by the instrument; **P < 0.01.

## Discussion

In-laboratory TAT is one of the quality indicators of testing^24^. Therefore, evaluating and improving in-laboratory TAT is essential for laboratory management^25,26^. There is considerable evidence that the factors influencing TAT in different hospitals vary. Furthermore, there is no unified evaluation method, and the traditional brainstorming method lacks objective empirical support.

In this study, we considered the dependent variable (whether TAT exceeded 30 min in the potassium test) and divided the data into timeout and non-timeout groups. We found that the influencing factors of untimely reporting of potassium included the number of items in the portfolio, specimen processing time, critical value ratio, instrument failure rate, shift, specimen status, and work experience. The Exp(B) value, where a positive B value indicates a delay in reporting, revealed that, except for work experience, the other factors could easily cause reporting delays. However, combined with multi-factor analysis, the highest risk was observed in group G (P < 0.05, OR = 14.47, Exp(B) = 2.67.

Considering the need for rotational training of young staff in various groups, the discontinuous and short work experience in the emergency department resulted in unskilled emergency work and TAT delays. In response, we extended the original 3-month emergency laboratory rotation period to 1 year and formulated a training schedule to conduct personnel assessments in the first week, first month, and first quarter of the emergency laboratory experience. This included the operation of various instruments in the emergency laboratory and the processing of reports. In group A (employees with over 30 years of work experience), the overtime rate was high (70.3%), and the overtime rate of all employees was more than 60%. Therefore, to increase the attention of employees to the blood potassium report TAT, we currently publish the blood potassium report TAT of the previous month at the beginning of each month. Additionally, we implemented a reward system for those who reported the lowest overtime rate in the current month. Following these improvements, the reported overtime rate of all groups decreased significantly, with the average overtime rate decreasing from 66.9 to 11.8% (P < 0.05). Specifically, the overtime rate of group G decreased from 69.2 to 11.7% (P < 0.05).

The second influencing factor was the high failure rate of the instruments (P < 0.05, OR = 1.46, Exp(B) = 0.38), which included biochemical detectors, centrifuges, and computer LIS. Considering the 24-h operation of the emergency laboratory, the fact that the laboratory had only a single biochemical module and centrifuge, both outdated and prone to faults after long-term operation, was an important issue. Thus, biochemical modules and centrifuges were added to extend the instrument’s lifespan, and computer and software systems were updated and upgraded to reduce network system lag, preventing disruptions to the report transfer. After these improvements, the failure rate of the instrument decreased from 45.3 to 0.1% (P < 0.05).

The final influencing factor was the length of specimen processing time (P < 0.05, OR = 1.13, Exp(B) = 0.13); specimen processing time is an important factor for TAT before specimen examination^22^. In this study, specimen processing time is the time between laboratory reception and instrumentation, including centrifugation and specimen transportation. During the centrifugation phase, centrifuges were added to ensure shorter waiting times, and timers with audible notifications were used as reminders to ensure the timely placement of specimens in the machine for inspection. Owing to changes in national policies, the peak of the new coronavirus has brought tremendous pressure to emergency laboratories. To cope with the increased volume of specimens, we have increased our emergency personnel to deal with the surge in the processing of other specimens. After these interventions, the specimen processing time decreased from 2317.2 ± 985.9 s to 1507.7 ± 535.2 s (P < 0.05).

Finally, the factors of acute potassium reporting timeout were analyzed using logistic regression analysis, and through targeted improvements, TAT decreased from 29 min (35–56) to 21 min (23–32) (P < 0.05) in the acute potassium test. The reporting timeout rate also decreased from 66.9 to 11.8% (P < 0.05), both of which are significant improvements.

Similar to other quality improvement studies, this study has limitations, including the single-center retrospective study design and small sample size. As this study was conducted in a single center, the results may not reflect the situation in other hospitals. Moreover, this study did not cover all aspects of the study factors, such as changes in the total number of specimens in the laboratory within 24 h. Therefore, we plan to address the above issues in our future research.

## Conclusion

The risk factors leading to delays in reporting emergency blood potassium were identified and addressed through quantitative statistics and logistic regression analysis. The reduction in the overtime rate of emergency blood potassium reporting confirmed the utility of logistic regression analysis in this field, which has rarely been reported previously. These findings provide an objective basis for the implementation of targeted interventions or preventive measures for quality management and novel suggestions for refined laboratory management.

## Data Availability

The datasets used and analyzed during the current study are available from the corresponding author upon reasonable request.
